# Humeral Shaft Fractures: Retrospective Cohort Comparing Operative Versus Nonoperative Management

**DOI:** 10.7759/cureus.93332

**Published:** 2025-09-27

**Authors:** Rodrigo A Beraldo, Caroline Izidorio Bernardes Silva, Isadora C Kobayashi, André A Picoli, Pedro M Rodrigues, Cassius A Ribeiro Filho, Ewerton Alexandre Galdeano, Renato Moraes

**Affiliations:** 1 Orthopaedics and Traumatology, Instituto Jundiaiense de Ortopedia e Traumatologia, Jundiai, BRA; 2 Orthopaedics and Traumatology, Hospital das Clínicas da Faculdade de Medicina da Universidade de São Paulo (University of São Paulo Faculty of Medicine Clinics Hospital), São Paulo, BRA; 3 Orthopaedics and Traumatology, Instituto Jundiaiense de Ortopedia e Traumatologia, Jundiaí, BRA

**Keywords:** functional bracing, humeral shaft fractures, plate osteosynthesis, quickdash, visual analog scale

## Abstract

Introduction: Operative fixation and functional bracing are established options for humeral shaft fractures, yet comparative patient-reported outcomes at one year remain debated. We compared 12-month function and pain between treatments.

Methods: We included 74 patients (36 treated surgically with plate fixation and 38 with functional bracing). Retrospective analysis of a prospective cohort (Hospital de Caridade São Vicente de Paulo, a tertiary center in Jundiaí, São Paulo, Brazil, January 2020-December 2021), including adults with AO 12 A2/A3/B2 diaphyseal fractures treated with plate osteosynthesis or functional bracing. Primary outcomes were QuickDASH and Visual Analog Scale (VAS) pain at baseline and 12 months. ANCOVA adjusted for baseline and age; a sensitivity model included complications.

Results: QuickDASH improved from 72.4±9.5 to 23.2±19.7 (Δ−49.2±18.3) with bracing and from 76.0±9.4 to 28.6±25.5 (Δ−47.4±21.9) with surgery, with between-group Δ p = 0.701. VAS fell from 6.4±1.2 to 1.8±2.1 and from 6.6±1.5 to 2.4±2.8, with between-group Δ p = 0.497. Adjusted 12-month differences (surgery−conservative) were QuickDASH +3.76 (95% CI −5.91 to +13.43; p = 0.446) and VAS +0.65 (−0.35 to +1.66; p = 0.202). Complications occurred in 19/74 (25.6%; 12 surgical, seven conservative): radial nerve neurapraxia 12/74 (all resolved ≤6 months), delayed union in three surgical (grafted at four months), and nonunion in four conservative (converted to surgery). In adjusted models, any complication independently worsened 12-month QuickDASH (+23.15; 95% CI +2.78 to +43.53; p = 0.026) but not VAS (+0.02; p = 0.979).

Conclusions: At 12 months, surgical fixation and functional bracing yield comparable patient-reported function and pain. Treatment should be individualized, while prevention and prompt management of complications may have a greater impact on functional recovery than the initial modality.

## Introduction

Humerus fractures account for approximately 1-3% of all fractures, and diaphyseal involvement occurs in about 20% [1-3]. The incidence is bimodal, predominating in young adult men and individuals over 60, with a higher prevalence among older women [4].

Both operative and conservative treatments are widely used and can yield satisfactory clinical outcomes. Conservative treatment with a Sarmiento functional brace is a simple option with good functional results, but has been associated with lower union rates than surgery [5,6]. Surgical management, most commonly intramedullary fixation or plate osteosynthesis, has gained prominence with advances in techniques and materials, including minimally invasive approaches, and has reported union rates around 90% with low rates of neurovascular injury or infection [7].

Indications remain debated, and fracture displacement, trauma energy, associated injuries, patient profile, and surgeon experience should be considered [8]. Without a definitive gold standard, treatment decisions are typically individualized after carefully appraising risks and benefits.

The primary objective of this study was to assess 12-month clinical outcomes, such as function and pain. The secondary objectives are to evaluate complications during follow-up and to perform subgroup analyses to explore potential effect modification.

## Materials and methods

Study design 

From January 2020 to December 2021, we conducted a retrospective analysis of a prospective cohort at Hospital de Caridade São Vicente de Paulo, a tertiary orthopedic and trauma center in Jundiaí, São Paulo, Brazil. Patients with diaphyseal humeral fractures were treated surgically or conservatively according to clinical judgment and patient preference.

The study was approved by the Institutional Research Ethics Committee of Jundiaí Medical School (approval no. 51062321.3.0000.5412) and conducted in accordance with the Declaration of Helsinki. All participants provided written informed consent before inclusion.

Eligibility

Inclusion criteria were age ≥16 years, presentation within 10 days of trauma, and AO 12 A2, 12 A3, or 12 B2 fractures [9]. Exclusion criteria were prior fractures/dislocations in the ipsilateral limb, Gustilo-Anderson [10] open fracture types II-III, pathological fractures, brachial plexus injury, cognitive impairment, active/previous infection in the affected limb, and inability to complete follow-up. Patients with cognitive impairment were excluded to ensure reliable reporting of patient-reported outcomes, as these instruments require adequate comprehension and self-reporting.

Treatment allocation 

For each eligible patient, the attending surgeon discussed the risks, benefits, and alternatives of operative fixation and conservative management with the patient and, when appropriate, family members. After all questions were addressed, treatment was selected through shared decision-making, with the final choice reflecting the patient's preferences and expectations.

Surgical treatment 

Three shoulder and elbow specialists operated using an anterolateral approach. Fractures were anatomically reduced and stabilized with a 4.5-mm narrow dynamic compression plate (DCP) using bicortical screws. Postoperatively, patients used an abduction pillow sling and followed a standardized rehabilitation protocol.

Conservative treatment

Patients were initially immobilized with a U-slab for two weeks, followed by a Sarmiento-type functional brace and a standardized rehabilitation protocol.

Rehabilitation protocol

The surgical group had a sling for four weeks, passive shoulder and elbow range of motion (ROM) initiated immediately after surgery and progressed as tolerated, active ROM from week 4, and strengthening from week 8.

The conservative group had a U-slab for two weeks, then Sarmiento functional brace; passive shoulder and elbow ROM initiated after brace fitting; active ROM from week 6; and strengthening from week 12 or earlier upon radiographic union.

Variables and measurements 

At enrollment, we recorded demographics (age, sex), side and dominance, and comorbidities (e.g., hypertension, diabetes). Patients were followed clinically and radiographically.

Patient-reported outcomes 

Upper-limb function was measured with the Quick Disabilities of the Arm, Shoulder and Hand (QuickDASH), an 11-item instrument scored on five-point Likert scales and transformed to a 0-100 score according to the official scoring instructions (higher scores indicate greater disability) [11]. Pain intensity was assessed with a 0-10 Visual Analogue Scale (VAS), a validated approach for subjective pain measurement [12,13]. Both instruments were administered in their standard forms without modification, and we did not reproduce item content in the manuscript.

Radiographic assessment 

Standard anteroposterior (AP) and lateral radiographs of the humerus were obtained at two, six, 12 weeks and six and 12 months. Images included the entire humeral diaphysis (and implant/brace when applicable) in two orthogonal projections per departmental protocol. Radiographic union was defined a priori as bridging callus across at least three of the four cortices on the AP and lateral views at the fracture site; union status was recorded at each scheduled visit.

Outcomes 

The primary outcomes were 12-month function (QuickDASH) and pain (VAS), both measured at hospital admission (baseline) and at 12 months. Predefined subgroup descriptors included age and the occurrence of clinical complications.

Complications

Complications were predefined and recorded prospectively from treatment initiation through 12 months using clinic notes and serial radiographs. Complications included radial nerve palsy, infection requiring antibiotics and/or surgery, delayed union (lack of expected radiographic progression by ~16-24 weeks), nonunion (absence of union by ≥6 months without progressive healing), implant-related failure (loosening, breakage, loss of fixation), conversion to surgery in the nonoperative group, and unplanned reoperation related to the index fracture or its treatment. For analysis, complications were summarized at the patient level as a binary variable (any complication: yes/no) and entered as a covariate in the adjusted 12-month ANCOVA; an event-level descriptive breakdown is also provided.

Statistical analysis 

Baseline characteristics were summarized as mean ± SD or median (IQR) and compared using the Mann-Whitney U test for continuous variables (e.g., age) and Fisher’s exact (2 × 2) or chi-square (>2 levels) tests for categorical variables. For unadjusted outcomes (QuickDASH and VAS), we described baseline and 12-month values within each treatment group, tested within-group change (12 months − baseline) using the Wilcoxon signed-rank test on paired data, and compared change between groups using the Mann-Whitney U test. 

Adjusted 12-month analyses used ANCOVA with HC3 robust standard errors, including the baseline score as a fixed covariate; in a separate model, we adjusted for (i) age (years) and (ii) complications up to 12 months (yes/no).

For transparency, parametric counterparts (paired t for within-group change and Welch’s t for between-group change; χ²/Fisher for categorical baseline variables) are also reported in the tables alongside p-values.

Both parametric and non-parametric tests were reported to enhance transparency and comparability with previous literature. Although the data did not strictly meet normality assumptions, presenting parametric results allows easier interpretation and consistency with prior studies.

## Results

The analytic sample comprised N = 74 participants (surgical n = 36, conservative n = 38). Age differed between groups (p = 0.039), whereas no other baseline variables showed statistically significant differences (several global tests with p > 0.999). Baseline characteristics are shown in Table 1.

**Table 1:** Baseline characteristics of the surgical and conservative groups. Between-group comparisons use Welch’s t-test for age and chi-square tests for categorical variables (df indicated). Two-sided α = 0.05; when tests were ≈1, p is reported as p > 0.999.

Variable	Surgical (n = 36)	Conservative (n = 38)	Test statistic	p
Age (Years)	36.1 ± 15.6	44.5 ± 18.5	Welch t = −2.102 (df≈71.0)	0.039
Sex (Female/Male)	14 (38.9) / 22 (61.1)	23 (60.5) / 15 (39.5)	χ² = 2.651 (1)	0.104
Affected side (Right/Left)	17 (47.2) / 19 (52.8)	23 (60.5) / 15 (39.5)	χ² = 0.836 (1)	0.360
Dominant side (Right/Left)	30 (83.3) / 6 (16.7)	33 (86.8) / 5 (13.2)	χ² = 0.009 (1)	0.923
Smoking (Yes/No)	5 (13.9) / 31 (86.1)	4 (10.5) / 34 (89.5)	χ² = 0.007 (1)	0.931
Alcohol use (Yes/No)	2 (5.6) / 34 (94.4)	2 (5.3) / 36 (94.7)	χ² ≈ 0.000 (1)	p>0.999
Diabetes (Yes/No)	5 (13.9) / 31 (86.1)	6 (15.8) / 32 (84.2)	χ² ≈ 0.000 (1)	p>0.999
Hypertension (Yes/No)	6 (16.7) / 30 (83.3)	9 (23.7) / 29 (76.3)	χ² = 0.213 (1)	0.645
Osteoporosis (Yes/No)	1 (2.8) / 35 (97.2)	2 (5.3) / 36 (94.7)	χ² ≈ 0.000 (1)	p>0.999

The most frequent comorbidities were hypertension (20%), diabetes (16%), and smoking (12%). Other conditions were uncommon.

Unadjusted pre- to 12-month changes are summarized in Tables 2-3. For QuickDASH, the conservative group decreased from 72.4 ± 9.5 at baseline to 23.2 ± 19.7 at 12 months (p < 0.001; Δ −49.2 ± 18.3), and the surgical group from 76.0 ± 9.4 to 28.6 ± 25.5 (p < 0.001; Δ −47.4 ± 21.9). The between-group comparison of change showed no statistical difference (p = 0.701). For pain (VAS), the conservative group decreased from 6.4 ± 1.2 to 1.8 ± 2.1 (p < 0.001; Δ −4.6 ± 2.1), and the surgical group from 6.6 ± 1.5 to 2.4 ± 2.8 (p < 0.001; Δ −4.2 ± 2.3), with no between-group difference in change (p = 0.497).

**Table 2:** Unadjusted QuickDASH: baseline versus 12 months by group. Between-group change (S−C): Welch t = 0.386 (df ≈ 68.4), p = 0.701. Values are mean ± SD. Within-group comparisons use paired t-tests; between-group comparison of change uses Welch’s t-test (df indicated). Two-sided α = 0.05; when tests were ≈1, p is reported as p > 0.999.

Group	Baseline	12 months	Δ 12m − baseline	Paired t (df)	p (within)
Conservative	72.4 ± 9.5	23.2 ± 19.7	−49.2 ± 18.3	−16.557	<0.001
Surgical	76.0 ± 9.4	28.6 ± 25.5	−47.4 ± 21.9	−13.008	<0.001

**Table 3:** Unadjusted VAS: baseline versus 12 months by group. Between-group change (S−C): Welch t = 0.683 (df ≈ 68.7), p = 0.497. Values are mean ± SD. Within-group comparisons use paired t-tests; between-group comparison of change uses Welch’s t-test (df indicated). Two-sided α = 0.05; when tests were ≈1, p is reported as p > 0.999.

Group	Baseline	12 months	Δ 12m − baseline	Paired t (df)	p (within)
Conservative	6.4 ± 1.2	1.8 ± 2.1	−4.6 ± 2.1	−13.605	<0.001
Surgical	6.6 ± 1.5	2.4 ± 2.8	−4.2 ± 2.3	−10.928	<0.001

After adjustment for age and baseline scores, no statistically significant between-group differences were observed at 12 months (Table 4). For QuickDASH, the adjusted mean difference (surgical − conservative) was +3.76 (95% CI −5.91 to +13.43; p = 0.449). The adjusted mean difference for VAS was +0.65 (95% CI −0.35 to +1.66; p = 0.206).

**Table 4:** Adjusted treatment effect at 12 months (ANCOVA, HC3). Analysis of covariance (ANCOVA) with heteroskedasticity-consistent (HC3) standard errors; F-test for the treatment group factor. Two-sided α = 0.05.

Outcome	Adjusted difference (S − C)	95% CI	F (df1, df2)	p
QuickDASH	+3.76	−5.91 to +13.43	0.580 (1, 70)	0.449
Visual Analog Scale (VAS)	+0.65	−0.35 to +1.66	1.626 (1, 69)	0.206

In secondary analyses, group × age interaction terms were insignificant for QuickDASH (p = 0.171) or VAS (p = 0.182). When complications were included as a covariate, their presence was associated with higher QuickDASH at 12 months (+23.15; 95% CI +2.78 to +43.53; p = 0.027), with no association for VAS (+0.02; 95% CI −1.74 to +1.79; p = 0.979) (Table 5).

**Table 5:** Association of complications with 12-month outcomes in adjusted models. Analysis of covariance (ANCOVA) including treatment group, baseline outcome, age, and any complication (yes/no) as covariates; F-test for the complication factor. Two-sided α = 0.05.

Outcome	Effect of complications (Yes vs No)	95% CI	F (df1, df2)	p
QuickDASH	+23.15	+2.78 to +43.53	5.101 (1, 69)	0.027
VAS	+0.02	−1.74 to +1.79	0.001 (1, 68)	0.979

Nineteen complications were identified (25.6%), 12 in the surgical group and seven in the conservative group. Radial nerve neurapraxia was the most frequent event (n = 12), diagnosed at presentation in eight patients (five surgical, three conservative) and postoperatively in four patients; all cases entirely resolved within six months. In the surgical group, three patients developed delayed union and underwent bone grafting at four months. In the conservative group, four patients progressed to nonunion (lack of union at six months) and were converted to surgery with bone grafting and fracture fixation.

## Discussion

In this cohort, baseline characteristics were broadly comparable between groups, except age, which was higher in one group (p = 0.039); other baseline variables showed no statistically significant differences (several global tests with p > 0.999). We therefore report unadjusted results first, followed by adjusted analyses that account for prognostic imbalance. 

In the unadjusted analysis, both groups improved substantially from baseline to 12 months, and the mean changes did not differ between treatments (QuickDASH Δ −49.2 ± 18.3 vs −47.4 ± 21.9; p = 0.987; VAS Δ −4.6 ± 2.1 vs −4.2 ± 2.3; p = 0.591), indicating no short-term between-group advantage in raw pre-post gains. This pattern aligns with randomized evidence from the FISH trial, where internal fixation did not yield superior 12-month functional outcomes compared with functional bracing, despite early advantages that faded by one year [14]. By contrast, the multicenter HUMMER cohort reported earlier recovery in the operative group. It concluded overall superiority for primary osteosynthesis at one year, with lower nonunion and better shoulder/elbow range of motion, highlighting that observational datasets may show more pronounced surgical benefits, particularly when structural complications are included [15]. Meta-analyses help reconcile these signals: van de Wall et al. [16] and Lode et al. [17] found that surgery reduces nonunion but does not consistently improve patient-reported function at the 12-month horizon, a synthesis consistent with our unadjusted equivalence in QuickDASH and VAS. Recently, Li et al. [18] emphasized faster early recovery with operation and lower nonunion/reintervention. Meanwhile, also noted that longer-term patient-reported outcomes tend to converge, again compatible with our short-term, unadjusted findings.

The age-adjusted primary analyses showed no statistically significant between-group differences at 12 months. The adjusted mean difference for QuickDASH (surgical − conservative) was +3.76 ( p = 0.446), and for VAS was +0.65 (p = 0.202); group × age interaction terms were not significant for either outcome. These results align with the 12-month convergence reported in randomized data and with meta-analyses that find no consistent advantage for one pathway in short-term patient-reported outcomes despite lower nonunion with surgery [16,17,19]. The discrepancy with studies favoring surgery on clinician-reported scales likely reflects differences in metrics and follow-up windows [20].

When complications were included as a covariate, their presence was independently associated with worse short-term function: QuickDASH was +23.15 points higher in patients with any complication (p = 0.026), whereas the association with VAS was null (p = 0.979). Descriptively, complications occurred in 25.6% of patients (12 surgical, seven conservative), most commonly radial nerve neurapraxia, resolving within six months; delayed union in three surgical cases was treated with grafting at four months, and four conservative nonunions (defined at six months) were converted to surgery with grafting and fixation. External evidence shows similar patterning of benefits and risks: bracing carries higher revision/nonunion/malunion, whereas surgery carries higher infection and radial nerve injury [20]; meta-analyses likewise report fewer nonunions but more deep infections with surgery [16,17], and technique/approach meaningfully shape neurological and infectious risks [21]. Our data and the literature suggest that preventing and promptly managing complications, as well as tailoring operative technique and approach, may influence short-term function more than the initial choice between operative and nonoperative pathways.

This study has limitations that inform interpretation. The nonrandomized, single-center design introduces selection bias and residual confounding despite prespecified adjustment for age and baseline outcomes; unmeasured factors such as detailed displacement/soft-tissue condition, fitness for surgery, and patient preferences may have influenced both treatment selection and recovery. The sample size was moderate, limiting precision for interaction testing and estimates within complication subtypes. Patient-reported outcomes, although administered by an independent research assistant, remain subject to reporting bias; rehabilitation adherence was not systematically tracked; radiographic union (bridging callus in at least three of four cortices) was assessed in routine care without inter-rater reliability testing; and crossover occurred when nonoperative cases were converted to surgery for nonunion. Significantly, follow-up was restricted to the short-term horizon of 12 months, which precludes conclusions about mid- or long-term outcomes such as durability of functional gains, late reoperations, implant-related problems, or delayed degenerative changes. Generalizability may be constrained to similar institutions and protocols. 

Residual confounding is possible, as we did not systematically capture fracture displacement, soft-tissue status, or adherence to rehabilitation, which may influence outcomes despite statistical adjustment.

## Conclusions

In this cohort of humeral shaft fractures, operative fixation and functional bracing yielded comparable 12-month outcomes after baseline age and scores adjustment, with no statistically significant between-group differences in QuickDASH or VAS. Treatment effects did not vary meaningfully with age, whereas complications were independently associated with worse functional status at 12 months.

These findings support an individualized approach to treatment selection, considering fracture pattern, soft-tissue status, comorbidities, patient preferences, and surgeon expertise, while prioritizing strategies that prevent and promptly address complications. Future prospective studies, adequately powered and with standardized capture of complication subtypes, rehabilitation adherence, and longer-term outcomes, are warranted to confirm these results and to identify patients most likely to benefit from each management pathway.
